# Theorizing bioarchaeology. By Pamela L. Geller, 2021. Springer, bioarchaeology and social theory series, 148 pp. ISBN: 978‐3‐030‐70702‐6. $140/$109 (hardback/e‐book)

**DOI:** 10.1002/ajpa.24528

**Published:** 2022-05-03

**Authors:** Liv Nilsson Stutz

**Affiliations:** ^1^ Department of Cultural Sciences Linnaeus University Växjö Sweden

Despite a long tradition of American four field anthropology with the promise of transdisciplinary thinking, and the significance of the bio‐cultural perspective in fields like public health, medical anthropology and medical humanities, the impact of the theoretical traditions informing cultural anthropology and archeology has been surprisingly limited in the fields of biological anthropology and bioarchaeology. One reason for this might be gatekeeping between the natural and cultural/social sciences, but in addition, there may also be a pervasive perception that theory is difficult, oblique, or perhaps irrelevant for biological anthropology and bioarchaeology, fields that both have tended to remain on the natural science side of the interdisciplinary equation. It is therefore very uplifting to see the publication of Pamela L. Geller's book *Theorizing Bioarchaeology* that builds a bridge between the disciplines in a manner that is both accessible and highly relevant.

The book takes on the issue of bridging social and critical theory with bioarchaeology in a very broad sense. It is organized into six chapters that in different ways theorize concepts, ideas, and perceptions in biological anthropology. It discusses how they in complex and entangled ways have come to affect bioarchaeology, and, most importantly, how the new perspectives that emerge from an engagement with critical theory, affect our understanding of the past and present. What I especially appreciate is the way in which Geller—across all themes discussed—demonstrates that theory must be deployed as a tool for the entire research process, and not act as a simple add‐on to provide depth or shine to the interpretation.

The first chapter “What Is Theorizing?” takes on several fundamental categories, concepts, and practices. It provides a genealogy of many of them and calls them out as limited cultural constructs that tend to project Western 19th century cultural expectations onto the universal. She introduces Donna Haraway's slashing of nature/culture and her subsequent merging of the two into “nature culture” and encourages us to “disrupt the dualisms altogether” (p. 5). This sounds more novel than it really is. Many could rightfully argue that it is the foundation for the bio‐cultural approach in anthropology, but perhaps the reference to Haraway still signals a shift in attitudes toward a more systematic engagement with social and critical theory.

The following three chapters (Chapter 2 “What Is Habitus?”, Chapter 3 “What is Normal?”, and Chapter 4 “What is Intersectionality?”) all demonstrate, through a tacking back and forth between critical theory and concrete bioarchaeological examples, the potential of combining the two. Her chapter on *habitus* connects the ideas of Marcel Mauss and Pierre Bourdieu to biomechanics, plasticity, and the implications on bioarchaeological data. It is pedagogical, engaging, and accessible as she explores the implications of practices such as walking in high heels. In her discussion on “what is normal” she shines as she demonstrates the implicit sexism in a discipline through concrete examples, like when we pathologize the female body and its role in reproduction but associate the male affliction of “atalatl elbow” as signaling ability. In addition to the usual suspects, in particular Foucault, but also Merleau‐Ponty (when discussing embodied experience), this chapter also introduces Georges Canguilhem as a thinker whose work on the body has the potential of becoming central for this new bioarchaeology. Similarly, the chapter on intersectionality provides a very useful introduction to the concepts. But the detailed Cuban case study on enslavement, emancipation, and race seems stylistically dislocated from the previous parts of the book. The case study breaks away from the earlier style of tacking back and forth between theories and applications, and it almost feels like a text that could have been published separately. I like it when the author takes on a more personal tone in the narrative, but it becomes a problem when these stylistic moments are not distributed mote equally throughout the work.

To sum up this first part of the book it is interesting to note that Chapters 1–4 all in different ways approach bioarchaeology as a form of life science. As a burial archeologist, I wonder where death is in all of this.

However, death is not completely absent. It enters the book in Chapter 5 “What Is Necropolitics?” This chapter centers on the work of Achile Mbebe with reference to Michel Foucault, Alberto Ciria, Giorgio Agamben, Katherine Verdery, and others to theorize violence, genocide, and the agentive corpse. While drawing on Mbembe, Geller also criticizes his dismissive view of archology as an apparatus of necropolitics itself, and instead highlights the work of archeology confronting and analyzing this violence (including Jane O'Dell, Jason de León, Alfredo González‐Ruibal, and others). Her defense of the present is not an excuse of the past. Geller critically reviews the role of biological anthropology and its dark history by examining foundational practices such as skull collecting in the past as well as currently used textbooks, highlighting the often unconscious reproduction of systemic violence in our discipline.

Finally, Chapter 6 “What Is Bioethos?” proposes a way forward toward a more ethical bioarchaeology that in many ways builds on the theoretical insights provided in previous chapters. It starts out firmly grounded in a North American experience discussing NAGPRA but evolves to consider race, class, gender, and religion, and to take on a range of emerging ethical conundrums for the discipline including the ethics of digitization and of ancient DNA, to the globalization of a discipline that still is very scattered in terms of priorities and values which makes the establishment of a broader code of ethics challenging. In the face of this complex challenge, Geller encourages us to engage in a proactive ethical practice of our discipline and develop moral normative practices in our interactions with the dead and the living. Drawing on an understanding of practice theory, she underlines the importance of understanding that these practices participate in forming our epistemological frames and ontological positions. She also embraces the entangled character of the responsibilities and accountabilities in our way (with reference to Karen Barad). “A bioethos,” Geller writes “is only imaginable if we seek to consciously entangle—materiality and discourse, words and deeds, knowing and being and doing, science and humanities, fact and compassion.”

This is a very ambitions book. It sets out to theorize bioarchaoelogy by casting a large net to capture the foundational critical theory and the history of the discipline, to provide theoretical tools for analysis and interpretation, and to inspire to engage in contemporary issues of social injustice and inequality. As if that was not hard enough, it also aims at finding a tone that is both pedagogical and engaging enough to capture the interest of students in the field and give them the tools to understand, and perhaps even love critical theory. Overall, I believe it succeeds.

I have a few reservations. My first critique is that the book approaches bioarchaeology like medical anthropology applied to the past, and it still asks questions mostly within the range of the living body, its use and health. What I am missing is the piece that pushes the theory further into asking questions about the domains more commonly associated to cultural anthropology and theoretical archeology, for example, ritual practice. When death is discussed in the book it is as something violent and extraordinary. It means that the author, while completely focused on concepts such as *habitus* and *hexis*, and while problematizing and exploring the concepts of normality and theorizing the lived experiences through the body, still does not incorporate death as a recurrent even crucial human experience for bioarchaeology to address. Here the work on the handling of the dead body as a ritualized practice would have provided an important addition. A second critique is that the book is clearly written for an U.S. audience. From a pedagogical point of view this makes sense, and this book will very likely become a core refence across U.S. campuses in the coming years. However, this results in a narrow scope, and misses the opportunity to address the ethics of bioarchaeology in an international perspective. When repatriation is discussed as an international phenomenon Canada is the only example. Similarly, the references to ongoing debates appear to be very anchored mostly in American scholarship (e.g., when discussing the debate about ancient DNA, most of the critically engaging and theorizing debates from European archeology are missing). This is relevant to point out since other parts of the work eloquently addresses issues such power, control of the discourse, disciplinary hegemony, and so forth.

These few reservations aside, this is an excellent book. I expect it will be given the attention it deserves in every U.S. anthropology classroom in the coming years, where I believe it has the potential of reshaping the next generation of bioarchaeologists into becoming more engaged with critical theory. If we agree with the argument of the author, and I do, this will not only result in a better understanding of lived experience in the past, but will also make us more ethical and aware as anthropologists.

## Data Availability

Data sharing not applicable to this article as no datasets were generated or analysed during the current study. This is a book review and none of the above applies.

